# Comparison of the efficacy and safety of 2% lidocaine HCl with different epinephrine concentration for local anesthesia in participants undergoing surgical extraction of impacted mandibular third molars: A multicenter, randomized, double-blind, crossover, phase IV trial

**DOI:** 10.1097/MD.0000000000006753

**Published:** 2017-05-26

**Authors:** Myong-Hwan Karm, Fiona Daye Park, Moonkyu Kang, Hyun Jeong Kim, Jeong Wan Kang, Seungoh Kim, Yong-Deok Kim, Cheul-Hong Kim, Kwang-Suk Seo, Kyung-Hwan Kwon, Chul-Hwan Kim, Jung-Woo Lee, Sung-Woon Hong, Mi Hyoung Lim, Seung Kwan Nam, Jae Min Cho

**Affiliations:** a Department of Dental Anesthesiology; b Department of Dental Anesthesiology and Dental Research Institute, Seoul National University School of Dentistry; c Department of Oral & Maxillofacial Surgery, Yonsei University College of Dentistry, Seoul; d Department of Dental Anesthesiology, Dankook University College of Dentistry, Dankook University, Cheonan-si, Chungnam; e Department of Oral and Maxillofacial Surgery, School of Dentistry, Pusan National University and Institute of Translational Dental Sciences, Pusan National University; f Department of Dental Anesthesia and Pain Medicine, School of Dentistry, Pusan National University and Dental Research Institute, Pusan National University Dental Hospital, Yangsan, Gyeongnam; g College of Dentistry, Wonkwang University, Iksan city, Jeonbuk; h Department of Oral and Maxillofacial Surgery, Dankook University College of Dentistry, Dankook University, Cheonan-si, Chungnam; i Department of Oral & Maxillofacial Surgery, Kyung Hee University Dental Hospital Kyung Hee University School of Dentistry, Seoul; j R&D Center, Huons Co. Ltd., College of Pharmacy, Hanyang University, Ansan-si, Kyeonggi-do, Republic of Korea.

**Keywords:** epinephrine, hemodynamics, lidocaine, local anesthesia, third molar

## Abstract

**Background:**

The most commonly impacted tooth is the third molar. An impacted third molar can ultimately cause acute pain, infection, tumors, cysts, caries, periodontal disease, and loss of adjacent teeth. Local anesthesia is employed for removing the third molar. This study aimed to evaluate the efficacy and safety of 2% lidocaine with 1:80,000 or 1:200,000 epinephrine for surgical extraction of bilateral impacted mandibular third molars.

**Methods:**

Sixty-five healthy participants underwent surgical extraction of bilateral impacted mandibular third molars in 2 separate visits while under local anesthesia with 2% lidocaine with different epinephrine concentration (1:80,000 or 1:200,000) in a double-blind, randomized, crossover trial. Visual analog scale pain scores obtained immediately after surgical extraction were primarily evaluated for the 2 groups receiving different epinephrine concentrations. Visual analog scale pain scores were obtained 2, 4, and 6 hours after administering an anesthetic. Onset and duration of analgesia, onset of pain, intraoperative bleeding, operator's and participant's overall satisfaction, drug dosage, and hemodynamic parameters were evaluated for the 2 groups.

**Results:**

There were no statistically significant differences between the 2 groups in any measurements except hemodynamic factors (*P* >.05). Changes in systolic blood pressure and heart rate following anesthetic administration were significantly greater in the group receiving 1:80,000 epinephrine than in that receiving 1:200,000 epinephrine (*P *≤.01).

**Conclusion:**

The difference in epinephrine concentration between 1:80,000 and 1:200,000 in 2% lidocaine liquid does not affect the medical efficacy of the anesthetic. Furthermore, 2% lidocaine with 1:200,000 epinephrine has better safety with regard to hemodynamic parameters than 2% lidocaine with 1:80,000 epinephrine. Therefore, we suggest using 2% lidocaine with 1:200,000 epinephrine rather than 2% lidocaine with 1:80,000 epinephrine for surgical extraction of impacted mandibular third molars in hemodynamically unstable patients.

## 1 Introduction

An impacted tooth is one that either fails to develop in its natural location or is hindered from eruption by an overgrowth of soft tissue, compact bone, or neighboring teeth. The treatment for such an impacted tooth is extraction of either the obstructing interference or the tooth itself.^[1]^ The most commonly impacted tooth is the third molar (TM). An impacted, partially erupted, or fully erupted TM can remain asymptomatic for many years but can ultimately cause acute pain, infection, tumors, cysts, caries, periodontal disease, and loss of adjacent teeth.^[2]^ In addition, there are reports of relapse after orthodontic retention and orthodontic crowding caused by a retained or an impacted TM.^[3]^

Local anesthesia is employed for removing TMs. Local anesthesia is a reversible blockade of nerve conduction in a circumscribed area that produces loss of sensation.^[4,5]^ Lidocaine is the most commonly used local anesthetic agent in dentistry and has excellent efficacy and safety.^[6]^ The rare incidence of serious life-threatening hypersensitivity reactions associated with lidocaine is the biggest clinical advantage, due to the frequent use of local anesthesia in dentistry.^[4]^ However, the possible neurotoxicity when used in high concentration is an important limitation of its use.^[7,8]^

There are several benefits of the addition of a vasoconstrictor to a local anesthetic: improvement in the duration and quality of anesthesia,^[9]^ reduction of blood loss throughout the operation,^[10]^ reduction in the peak plasma concentration of the anesthetic agent,^[11,12]^ and decrease of the minimum concentration of anesthetic necessary for nerve block.^[13,14]^ Epinephrine is the most studied and widely used vasoconstrictor. Epinephrine produces its vasoconstrictor effects by binding to and stimulating α1-adrenergic receptors in the walls of arterioles.^[15]^ At low systemic concentrations usually employed in dental anesthesia, epinephrine can increase cardiac output, heart rate (HR), and peripheral vasodilation.^[16]^

Lidocaine concentration affects the efficacy and safety of local anesthesia in patients undergoing surgical extraction of an impacted mandibular TM.^[17,18]^ In addition, 2% lidocaine solution used for inferior alveolar nerve block in patients with symptomatic irreversible pulpitis had similar success rates when used with 1:80,000 or 1:200,000 epinephrine concentration.^[19]^ This suggests that the efficacy of 2% lidocaine solution will not differ when it is used with 1:80,000 or 1:200,000 epinephrine concentration.

However, the efficacy and safety of 2% lidocaine with 1:80,000 or 1:200,000 epinephrine in surgical extraction of bilateral impacted mandibular TMs has never been compared so far. Hence, we aimed in this study to evaluate the efficacy and safety of 2% lidocaine with 1:80,000 or 1:200,000 epinephrine in surgical extraction of bilateral impacted mandibular TMs.

## 2 Methods

### 2.1 Study oversight

The investigation was registered at http//www.clinicaltrials.gov (NCT 02696369) and performed according to the guidelines for the proper conduct of medical research on human participants. The study protocol was reviewed and approved by the research ethics boards of each participating hospital in Korea [Seoul National University Dental Hospital (CME 14002); Busan National University Dental Hospital (PNUDH-2014-001-MS); Kyung Hee University Dental Hospital (KHDIRB 1410-5); Dankook University Dental Hospital (H-1407/009/005); Yonsei University Dental Hospital (2-2014-0017); and Wonkwang University Dental Hospital (WKDIRB 201410-02)]. Written informed consent was obtained from all participants. All aspects of participant privacy and confidentiality were preserved. The study was conducted in accordance to the Declaration of Helsinki.^[20]^

### 2.2 Design

The trial was a multicenter, randomized, double-blind, crossover, phase IV trial.

### 2.3 Participants and study design

The study subjects included 65 participants with symmetrically located impacted mandibular TMs, as detected in panoramic radiographs. They were recruited by advertisements. The inclusion criteria were age over 19 years; physical grade 1 or 2 according to the American Society of Anesthesiologists (ASA);^[21,22]^ requirement of bilateral surgical extraction of impacted mandibular TMs (either mesioangular or horizontal angulation of Winter's classification) and similar degree of impaction in both sides; and subjects who agreed and signed written inform consent. The exclusion criteria were a history of hypersensitivity to lidocaine or this group of drugs; presence of active infection or abscess at the time of extraction; coagulation disorder, hyperthyroidism, atherosclerosis, heart failure, convulsions, uncontrolled hypertension, or diabetes mellitus; current use of vasoconstrictors, ergot alkaloids, phenothiazines, butyrophenones, tricyclic antidepressants, monoamine oxidase inhibitors, sedatives, or anxiolytics; use of anticoagulants or antiplatelets, including aspirin, systemic corticosteroids, or nonsteroidal anti-inflammatory drugs, within 7 days before the extraction date; use of analgesics within 24 hours before the extraction; requirement for sedatives or antianxiolytic drugs during the extraction; other operative plans requiring general or local anesthesia during the clinical trial period; other medical history that might affect the clinical trial (e.g., malignant tumor, immunodeficiency, kidney disease, liver disease, lung disease, or unstable psychiatric condition); pregnancy or breastfeeding; planned pregnancy or intention of using contraception during the clinical trial period; use of other investigated products or medical devices within 4 weeks before the extraction date; and a history of prior oral or maxillofacial surgery.

The statistician randomly assigned the participants using the block randomization method with SAS (SAS Institute, Cary, NC). The statistician delivered a list of random assignment codes to the pharmacy packager. 2% lidocaine with 1:200,000 epinephrine (2% Lidocaine HCL Injection, Huons Co., Ltd, Seongnam, Korea) and 2% lidocaine with 1:80,000 epinephrine (2% Lidocaine HCL:Epinephrin inj. , 3 M, Seoul, Korea) were packaged so that they could not be recognized and were distributed to the trial institutes. The participants were randomly assigned to receive 2% lidocaine with epinephrine at a concentration of 1:80,000 (the L80 group) or 1:200,000 (the L200 group) at a 1:1 ratio. This study was double blinded; neither the operator nor the participant was aware of which anesthetic was administered. Each participant required equal surgical care on opposite sides of the mandible, which was conducted in 2 visits, 1 to 4 weeks apart. At the first visit, the participant was randomly assigned to receive 2% lidocaine with 1 of the 2 concentrations of epinephrine (L80 or L200). At the second visit, the participant received 2% lidocaine with epinephrine at the concentration that was not used in the first visit. To minimize factors affecting the efficacy and safety evaluation of the trial drugs, all operations, measurements, and postoperative controls were performed by a trained operator in each research institute. Initially, the participants were administered extraoral antisepsis with 0.2% chlorhexidine gluconate and intraoral antisepsis with 0.12% chlorhexidine gluconate. Afterward, the participants received a regional anesthetic blockade of the inferior alveolar nerves and long buccal nerve infiltration with each 1.8 mL of the anesthetic solution. If the participant complained of pain during the extraction, an additional local anesthetic was administered, and the additional dosage was recorded in the case record. Extraction of the TM was performed by the buccal approach technique using rotation instruments.^[23]^ At the end of the operation, the operating sites were thoroughly irrigated, suctioned, and sutured.

### 2.4 Measurements

The following parameters were assessed. Efficacy variables were divided into primary outcome and secondary outcomes. The primary outcome was “visual analog scale (VAS) pain score measured immediately after surgical extraction.” The secondary outcomes were “The onset time of local anesthesia,” “Duration of analgesia,” “VAS pain score recorded 2, 4, and 6 hours after the administration of local anesthetics,” “The time interval between the administration of the local anesthetics and the first postoperative pain sensation,” “Perioperative bleeding assessment by the operator,” “Operator's overall satisfaction,” “Participant's overall satisfaction,” and “Drug dosage administered.” The safety variables were “Adverse event” and “Vital signs.” “VAS pain scores measured immediately after the surgical extraction” was measured by the VAS score. The VAS consisted of a horizontal row of light-emitting diodes of 100-mm length. The participants were told that the left end of the scale, labeled “minimum,” corresponded to “no pain at all,” and the right end, labeled “maximum,” corresponded to “maximum imaginable pain.”^[24,25]^ The participants indicated their level of pain and the operator recorded their response. “The onset time of local anesthesia” was assessed by the loss of sensibility of the lower lip, the corresponding half of the tongue, and the mucosa. “Duration of analgesia” was measured by the lack of sensibility of the lower lip, tongue, and mucosa. The participants recorded the moment that the anesthesia had worn off. “Drug dosage administered” was measured by the total volume (mL) of anesthetic solution used during the operation. “Perioperative bleeding assessment by the operator,” “operator's overall satisfaction,” and “participant's overall satisfaction” were measured by the Likert scale.^[26]^ The Likert scale scores 1–5 of “Perioperative bleeding assessment by the operator” were presented with 1 anchored by “a few bleeding” and 5 anchored by “very much bleeding.” The Likert scale 1–5 of “Operator's overall satisfaction” and “participant's overall satisfaction” were presented with 1 anchored by “very dissatisfied” and 5 anchored by “very satisfied.” “Adverse event” referred to an undesirable and unintended sign, symptom, or disease that occurred during the administration or use of a clinical trial drug. If an adverse event from the first visit continued to the second visit, it was not regarded as an adverse event resulting from the second visit. Any adverse event occurring before the extraction of the remaining mandibular TM was considered an adverse event resulting from the first visit. The frequencies and percentages of occurrence of adverse events and adverse drug reactions were calculated for each group. The severity of an adverse event was assessed by the following criteria. Mild: causes minimal inconvenience without interfering with the normal daily life (functioning) of the participant and is easily tolerated. Moderate: causes inconvenience that significantly interferes with the normal daily life (functioning) of the participant. Severe: makes daily life (functioning) of the participant impossible. “Vital signs” was the following: systolic, diastolic blood pressure (BP), and HR were measured by oscillometric BP measurement devices and pulse oximetry automatically and noninvasively. BP and HR were measured as follows: immediately before drug administration; up to 5 minutes after drug administration: 5 times every 1 minute; 5 minutes to 20 minutes after drug administration: 5 times every 3 minutes; 20 minutes after drug administration to 70 minutes: measured every 5 minutes.

### 2.5 Statistical analysis

Categorical variables are presented as absolute numbers and percentages. Means with standard deviations (SD) were used when the continuous variables followed the normal distribution, and medians with interquartile range were used when the continuous variables did not follow the normal distribution. The statistical significance of the difference in parameters within each group was determined by the paired *t*-test or by the Wilcoxon signed-rank test when the distribution was not normal. Adverse events were assessed with Pearson's χ^2^ test or Fisher's exact test to find the homogeneity between the 2 groups. Vital signs were assessed with the paired *t*-test or the Wilcoxon signed-rank test. A 2-tailed *P-*value of <.05 was considered to indicate a statistically significant difference. Analyses were performed using SPSS Statistics version 21 (SPSS, Chicago, IL).

### 2.6 Sample size calculation

The sample size calculation was based on an α-level type I error of 0.025 for a 1-tailed test, a β-level type II error of 0.1, and a statistical power of 90%. Following research on the efficacy of 4% articaine with 1:100,000 epinephrine versus 1:200,000 epinephrine,^[27–29]^ the VAS scores for postoperative pain, and considering a difference of 15 mm as clinically significant (estimated mean standard deviation, 27 mm), the sample size was calculated as 23 participants in each group. A dropout rate of 20% was assumed, and at least 29 participants were enrolled in each group. The upper limit of the VAS score was set at 15 mm to determine the noninferiority tolerance limit with a confidence interval of 97.5%. If the measurement is below the upper limit, the result for the L200 group is noninferior to the result for the L80 group.

## 3 Results

### 3.1 Participant flow

**Figure 1:**
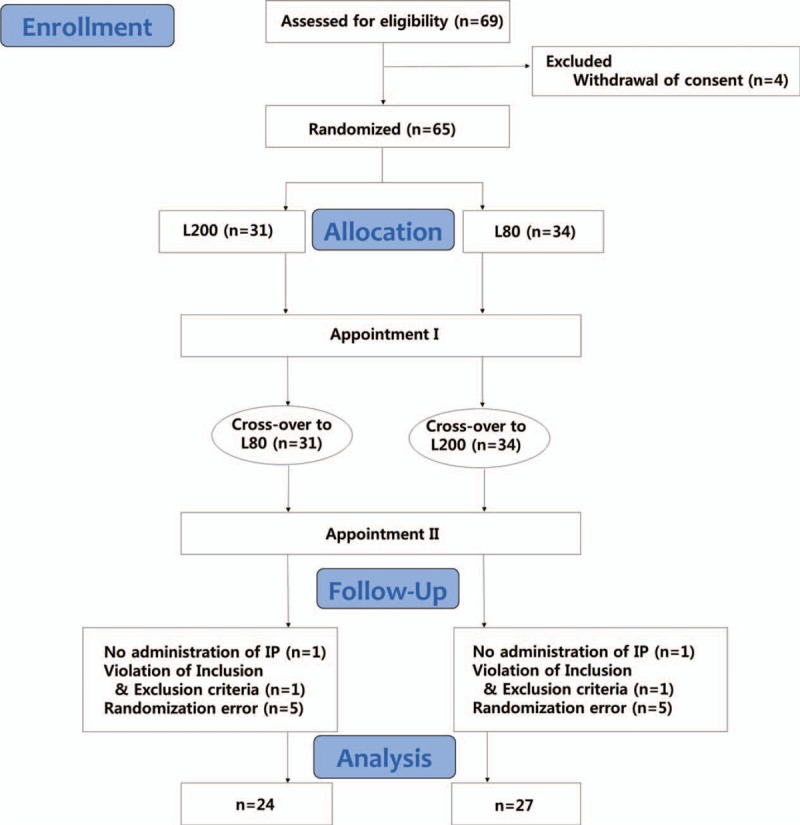
Flow chart of clinical trial procedure and randomization of double-blind, crossover study with all participants receiving both treatments. IP = investigational product, L80 = 2% lidocaine with 1:80,000 epinephrine, L200 = 2% lidocaine with 1:200,000 epinephrine, n = group size.

Sixty-nine participants were recruited. Sixty-five were eligible and were randomly assigned to the L80 or the L200 group. Fifty-one (78.5%) of the 65 participants completed the study, including 24 of 31 participants in the initial L200 group (77.4%) and 27 of 34 participants in the initial L80 group (79.4%). The participants underwent their first operation with each concentration of local anesthesia and underwent operation with the other concentrations of local anesthesia on the other side after 1 to 4 weeks. Ten participants dropped out because of randomization error. Four participants were omitted because the study drugs were not administered or the inclusion or exclusion criteria were violated. The total number of samples was 102, because 51 participants had anesthesia with 2 concentrations of epinephrine (Fig. 1). The baseline characteristics of the sample are depicted in Table 1.

**Table 1:**
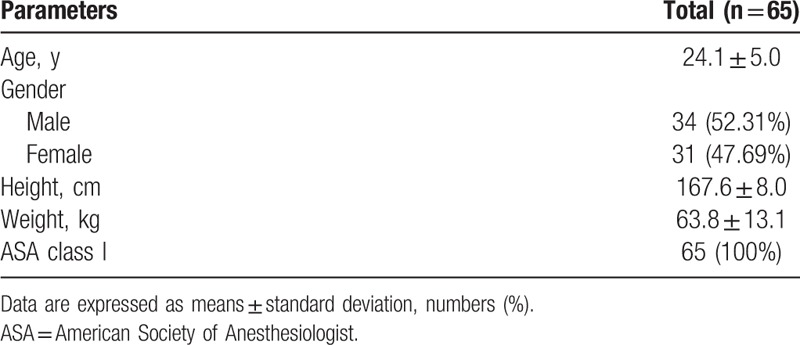
Demographic characteristics of the participants (n = 65).

### 3.2 Efficacy results

Pain experienced during surgical extraction was measured immediately after the operation by VAS. The mean ± SD VAS score was 13.7 ± 1.9 mm in the L80 group and 20.0 ± 2.5 mm in the L200 group (Table 2). Although the participants in the L200 group reported having experienced more intense pain than those in the L80 group (*P* = .055), the upper limit of the 1-sided confidence interval was 12.9 mm, which is lower than the noninferiority tolerance of 15 mm (Table 2). Therefore, the pain control of L200 was noninferior to that of L80. The secondary efficacy analysis was carried out to compare the time of onset of anesthesia for the 2 concentrations of epinephrine. The mean ± SD time of onset was 4.9 ± 4.1 minutes in the L80 group and 5.2 ± 4.1 minutes in the L200 group, with no significant difference between the groups (*P* = .447) (Table 3). The mean ± SD duration of action of anesthesia was 183.5 ± 5.0 minutes in the L80 group and 182.2 ± 5.4 minutes in the L200 group, with no significant difference between the groups (*P* = .758) (Table 3). There were no significant differences in the mean ± SD VAS pain scores between the 2 groups at 2, 4, and 6 hours after administration of the anesthetic (*P* >.05) (Table 3). The mean ± SD time taken for the participant to report postoperative pain was 255.5 ± 11.0 minutes in the L80 group and 237.5 ± 14.9 minutes in the L200 group, with no significant difference between the groups (*P* = .246) (Table 3). The mean ± SD bleeding tendency was 2.0 ± 0.1 in the L80 group and 2.2 ± 0.1 in the L200 group, with no significant difference between the groups (*P* = .206) (Table 3). The mean ± SD overall satisfaction score of the operators was 3.9 ± 0.9 in the L80 group and 3.8 ± 1.0 in the L200 group, with no significant difference between the groups (*P* = .548) (Table 3). The mean ± SD overall satisfaction score of the participants was 3.6 ± 0.1 in the L80 group and 3.7 ± 0.1 in the L200 group, with no significant difference between the groups (*P* = .693) (Table 3). The mean ± SD drug dose administered was 3.6 ± 0.1 in the L80 group and 3.6 ± 0.2 in the L200 group, with no significant difference between the groups (*P* = .163) (Table 3).

**Table 2:**
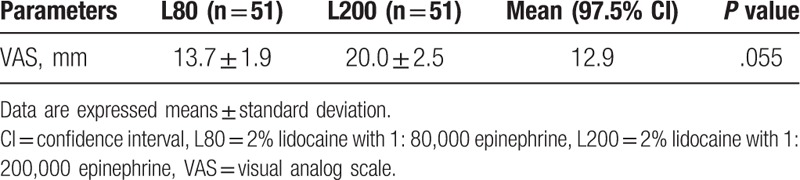
Comparison of VAS after the surgical extraction for L80 and L200 groups.

**Table 3:**
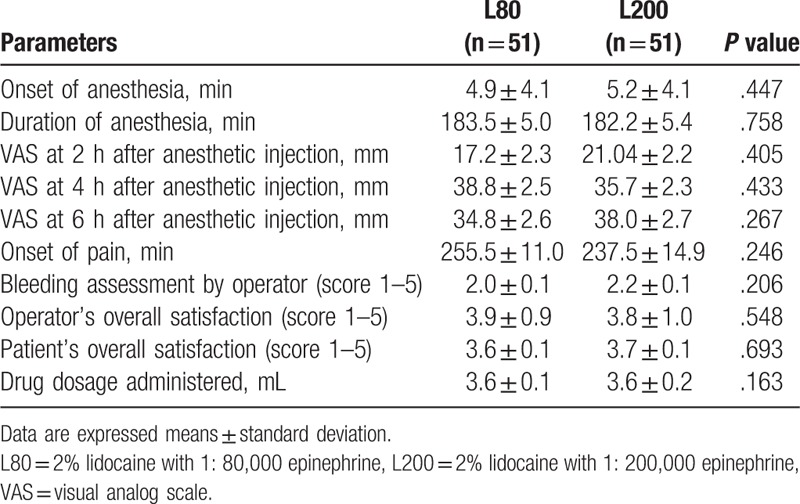
Comparisons of secondary outcomes after the surgical extraction for L80 and L200 groups.

### 3.3 Safety results

After administration of the anesthetic, adverse events at the extraction site occurred in 2 participants (3.1%) in the L80 group and 3 participants (4.7%) in the L200 group. Alveolar osteitis occurred in 4 participants, 2 from each group, and inflammation of the administration site occurred in 1 participant in the L200 group. There was no statistically significant difference between the groups in the frequency of these adverse events (*P* = 1.000). Adverse events away from the operating site occurred in 2 participants (3.1%) in the L80 group and 3 participants (4.7%) in the L200 group. These were diarrhea and headache in 1 participant each in the L80 group and lower abdominal pain, myalgia, and temporomandibular joint syndrome in 1 participant each in the L200 group. One mild and 1 moderate adverse event occurred in the L80 group. Of the 3 adverse events in the L200 group, 2 were considered mild and the other was considered moderate. There was no statistically significant difference between the groups in the frequency of these adverse events (*P* = 1.000).

Changes in the participants’ vital signs (systolic and diastolic BP and HR) from before and after administration of the anesthetics were analyzed. Vital signs measured after the administration of anesthetics were significantly increased compared with baseline measurements. In the L80 group, the changes in vital signs were systolic BP 14.1 ± 10.2 mm Hg (*P* <.001), diastolic BP –10.8 ± 12.9 mm Hg (*P* <.001), and HR 14.8 ± 11.1 (*P* <.001) (Table 4). In the L200 group, the changes in vital signs were systolic BP 9.3 ± 7 .3 mm Hg (*P* <.001), diastolic BP –8.4 ± 6.6 mm Hg (*P* <.001), and HR 10.5 ± 12.5 (*P* <.001) (Table 4).

**Table 4:**
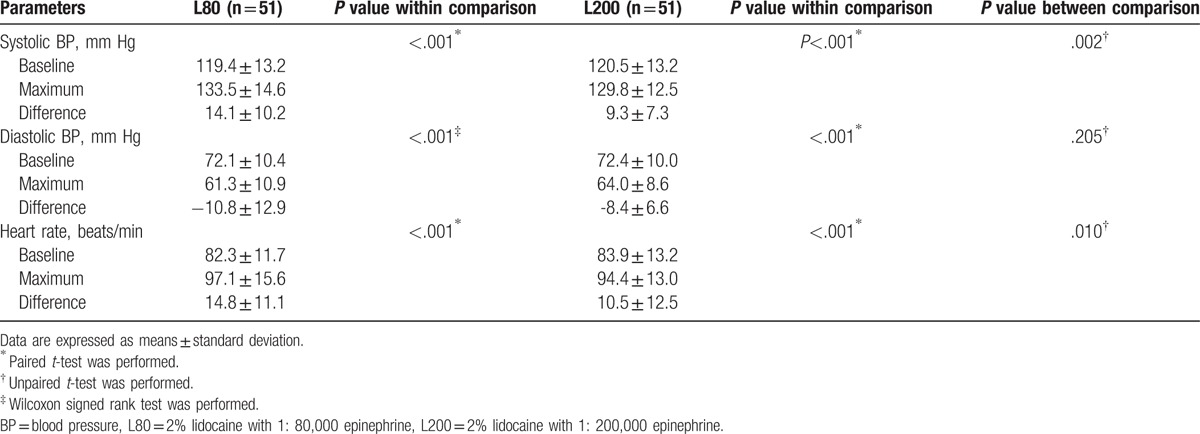
Comparisons of vital signs before and after the administration for L200 and L80 groups.

## 4 Discussion

The TM is the most commonly impacted tooth, and local anesthesia is necessary for treatment. Lidocaine is the most commonly used local anesthetic, and epinephrine is an excellent vasoconstrictor that is added to lidocaine.^[6,7]^ Efficacy and complications differ depending on the concentration of epinephrine added to lidocaine.^[9,15,16,30,31]^ Studies of the efficacy and safety of 2% lidocaine with 1:80,000 or 1:200,000 epinephrine for treatment of the impacted TM have not previously been conducted.

This study comparatively evaluated the efficacy and safety of 2% lidocaine with 1:80,000 or 1:200,000 epinephrine in surgical extraction of bilateral impacted mandibular TMs. Although there was a difference in VAS pain scores between the 2 groups after the operation, it was not statistically significant (*P* >.05) (Tables 2 and 3). There was also a difference between the groups in time of onset of pain after the operation, but it was also not statistically significant (*P* = .246) (Table 3). The changes in systolic BP and HR after the administration of the anesthetics were significantly greater in the L80 group than in the L200 group (*P* ≤.01) (Table 4). There were no severe adverse events resulting from the anesthetics.

Our limit for noninferiority tolerance of 15 mm is very strict, because 1 clinical study of articaine versus lidocaine for TM extraction reported that a difference of 20 mm in the VAS pain score was clinically significant, with a sample size of 20 patients in each group, a type II error of 0.2, a type I error of 0.05, and a statistical power of 80%.^[28]^ This is significant meaningful that there is no difference in VAS between the 2 groups in our study, despite the strict limit.

In dentistry, epinephrine is the most commonly used vasoconstrictor for local anesthesia to provide excellent anesthesia and bleeding control.^[9]^ Although epinephrine is an effective vasoconstrictor and is generally safe, it has many adverse effects depending on the dosage, such as hypertension, tachycardia, arrhythmia, and circulatory failure, especially in patients with cardiovascular diseases.^[30,31]^ Focusing on this point, clinical trials using 6 to 8 cartridges of the anesthetic solution with 1:100,000 epinephrine concentration found increases in BP and HR.^[32,33]^ In the present study, there were no differences between the 2 groups in adverse events after administration of the anesthetics. Therefore, epinephrine at 1:200,000 concentration is presumed to be equally effective as the 1:80,000 concentration and safer than the 1:80,000 concentration for patients with hemodynamically unstable patients.

A limitation of this study is that all participants were ASA class I. Since systolic BP and HR increased even in healthy participants, we can predict that vital signs would be unstable in unhealthy participants. However, this study could not proceed to unhealthy participants in ASA class III or higher because of ethical issues.

In conclusion, a difference in epinephrine concentration between 1:80,000 and 1:200,000 in 2% lidocaine liquid does not affect medical efficacy. Furthermore, 2% lidocaine with 1:200,000 epinephrine has better safety with regard to hemodynamic parameters than 2% lidocaine with 1:80,000 epinephrine. Therefore, we suggest using 2% lidocaine with 1:200,000 epinephrine in surgical extractions of impacted mandibular TMs rather than 2% lidocaine with 1:80,000 epinephrine in hemodynamically unstable patients. Further studies should be conducted in patients with cardiovascular disease because of a lack of studies to date on the use of 2% lidocaine with 1:200,000 epinephrine in this patient population.

## Acknowledgment

This work was supported by Huons Co., Ltd. Pharmaceutical Company, Republic of Korea.
